# Using electroacupuncture to recover muscle strength in patients with knee osteoarthritis after total knee arthroplasty: a study protocol for a double-blinded, randomized, and placebo-controlled trial

**DOI:** 10.1186/s13063-020-04601-x

**Published:** 2020-08-10

**Authors:** Hui Xu, Bingxin Kang, Yulin Li, Jun Xie, Songtao Sun, Sheng Zhong, Chenxin Gao, Xirui Xu, Chi Zhao, Guowei Qiu, Lianbo Xiao

**Affiliations:** 1 Department of Joint Orthopaedics, Guanghua Hospital Shanghai University of Traditional Chinese Medicine, Shanghai, 200050 China; 2 Shanghai University of Traditional Chinese Medicine, Shanghai, 201203 China; 3 Shanghai Guanghua Hospital of Integrated Traditional Chinese and Western Medicine, Shanghai, 200050 China; 4 Arthritis Institute of Integrated Traditional Chinese and Western Medicine, Shanghai Academy of Traditional Chinese Medicine, Shanghai, 200050 China

**Keywords:** Electroacupuncture, Knee osteoarthritis, Total knee arthroplasty, Study protocol

## Abstract

**Background:**

Total knee arthroplasty (TKA) is a gold standard for patients with terminal term gonarthrosis for reducing pain, correcting deformities, and regaining stability. However, post-TKA muscle strength recovery is often difficult. Although electroacupuncture (EA) enhances lower extremity muscle strength of the lower extremity, there is limited evidence regarding its effect on lower extremity muscle strength in post-TKA patients. Consequently, this trial intends to evaluate the efficacy of post-TKA EA on the recovery of lower extremity muscle strength, specifically, during the early post-TKA period.

**Methods/design:**

This is a double-blinded, randomized, and controlled trial. It will be conducted between August 2020 and December 2020. Ninety-four participants with KOA who have undergone unilateral TKA will be randomized into a treatment (EA) group and a control (sham EA) group. The former and latter groups will receive EA and sham EA, respectively, at ST37, ST36, SP10, and SP9 acupoints. The participants will undergo ten treatment sessions over 2 weeks (5 sessions per week). The primary outcomes will include changes in muscle strength and the Hospital for Special Surgery score at the second week from baseline (pre-op 1 day or POD 3). The secondary outcomes will include a 4-m walk test, numerical rating scale score, the Hamilton Anxiety Scale score, and additional analgesia use. Additional outcomes will include the incidence of analgesia-related side effects and the participant satisfaction rate. Participant blinding will also be assessed where they will be asked to guess whether they received EA after the latest intervention. Adverse EA events will be documented and assessed throughout the trial.

**Discussion:**

EA is helpful for post-TKA recovery and enhancement of lower limb muscle strength.

**Trial registration:**

Chinese Clinical Trial Registry ChiCTR1900027806. Registered on 29 November 2019

## Background

As a common chronic joint disease in orthopaedics, knee osteoarthritis (KOA) is mainly aetiologically caused by degenerative changes in the articular cartilage and the resulting hyperosteogeny. This results in complications, including knee pain, stiffness, and dysfunction. Given the improvements resulting from new biomaterials and surgical techniques, total knee arthroplasty (TKA) is now a first-choice treatment for patients with severe KOA for improving their quality of life [1, 2]. Compared with non-surgical treatment, TKA significantly relieves pain and promotes functional improvement in patients with severe KOA [3]. More than 700,000 TKA procedures are performed annually in the USA to relieve KOA-related pains and disability, which is expected to reach 3.5 million per year by 2030 [4]. Despite the objective post-TKA improvements in knee function and its imaging parameters, 19–23% of post-TKA patients still report dissatisfaction at the 6-month follow-up [5, 6]. Compared with healthy adults, there are post-TKA deficits in lower limb muscle strength, which reduces walking distance and stair climbing speed [7, 8]. Lack of muscle strength increases the risk of falling, which makes the patients lose their independence. Muscle strength rehabilitation of the knee extensor and flexor is essential for functional improvement of patients with KOA since knee stability is primarily supported by the soft tissue of the quadriceps femoris and hamstring. A post-TKA decrease in the strength of the quadriceps femoris and hamstring [9–12], which is associated with decreased function [13, 14], has been reported. Moreover, there have been reports of difficulty with post-TKA muscle strength recovery. For example, surgical incision pain reduces the patient’s ability to exercise. In the first post-TKA month, the strength of the quadriceps femoris decreased by 60% [15]. Although the strength of the quadriceps improves over time, approximately 30% of post-TKA patients have reported deficits at > 2 postoperative years compared with healthy adults [16]. Immediately after the operation, the hamstring strength has been reported to reduce by 50% [11, 17]. Previous studies have reported TKA-related weakness and gait biomechanical damage to the quadriceps femoris and hamstring that may last for several years [18, 19]. Standardized physiotherapy is beneficial towards muscle functional improvement [20]. Moreover, neuroregulatory techniques, including neuromuscular electrical stimulation and electroacupuncture (EA), have become increasingly popular as post-TKA rehabilitation therapy [21]. Effective post-TKA neuroregulatory techniques can enhance lower limb muscle strength, which fast-tracks rehabilitation and reduces the financial burden of patients [22].

EA is primarily recommended for pain modulation and has been shown to enhance muscle strength [23, 24]. However, there have been few randomized controlled trials on post-TKA patients with limited evidence regarding the EA effect on lower limb muscle strength. Therefore, we designed a double-blinded, randomized, and placebo-controlled trial to investigate the EA effects on lower limb muscle strength in post-TKA patients, specifically, during the early postoperative period.

### Objectives

To evaluate the efficacy of EA after TKA for enhancing the muscle strength of the lower extremity in the early postoperative period.

## Methods/design

This trial will be conducted at the Department of Joint Orthopaedics, Shanghai Guanghua Hospital of Integrated Traditional Chinese and Western Medicine, Shanghai, China, which is a teaching and tertiary hospital of Shanghai University of Traditional Chinese Medicine, Shanghai, China. Patients with KOA who will undergo unilateral TKA will be mainly enrolled from the orthopaedics wards of Shanghai Guanghua Hospital of Integrated Traditional Chinese and Western Medicine, Shanghai, China, through advertisements. We followed the SPIRIT 2013 Statement [25] and STRICTA [26] to report this trial protocol. This study has been approved by the ethics committee of Shanghai Guanghua Hospital of Integrated Traditional Chinese and Western Medicine (Ethics Approval Number: 2019-K-23) and registered at the Chinese Clinical Trial Registry. All participants will be asked to provide written informed consent and will be informed that the trial will not involve the collection of biological specimens for storage.

### Eligibility criteria

The inclusion criteria will be as follows: (1) 60–80 years of age, (2) diagnosed with KOA and willing to undergo unilateral TKA, (3) undergone similar surgical approach with normal blood coagulation function, (4) having posterior cruciate-stabilizing prostheses (Smith & Nephew, London, UK), (5) having well-placed knee prosthesis (as shown by the postoperative X-ray), and (6) postoperative clear consciousness and normal cognitive function.

The exclusion criteria will be as follows: (1) serious nervous system problems, cardiovascular diseases, osteoporosis, and metabolic diseases; (2) having coagulation disorder, haemophilia, or tumour; (3) intraoperative bone fracture, dislocation, and structural abnormality; and (4) skin damage in the acupoint area.

### Exit criteria and management

Exit criteria include (1) participant request, (2) severe postoperative complications (e.g. pulmonary embolism), and (3) intratreatment side effects.

### Sample size

The sample size will be calculated using the previously reported peak torque (PT) of the quadriceps femoris [27]. The formula is as follows:

$$ n=\frac{2{\left({Z}_{\alpha }+{Z}_{1-\beta}\right)}^2{\sigma}^2}{\Delta ^2} $$

An effect size (△) of 2 with a standard deviation (SD) of 3.34 between EA and RT (*n* = 33*2/0.85 = 82) will be adopted. Based on this data, a two-group trial will be conducted with 39 participants per group, *α* = 0.05 (two-sided), and *β* = 0.2 (80% power) [28]. With a possible sample loss of 15%, the final sample size will be set to 47 participants per group.

### Recruitment strategies and enrollment

**Fig. 1:**
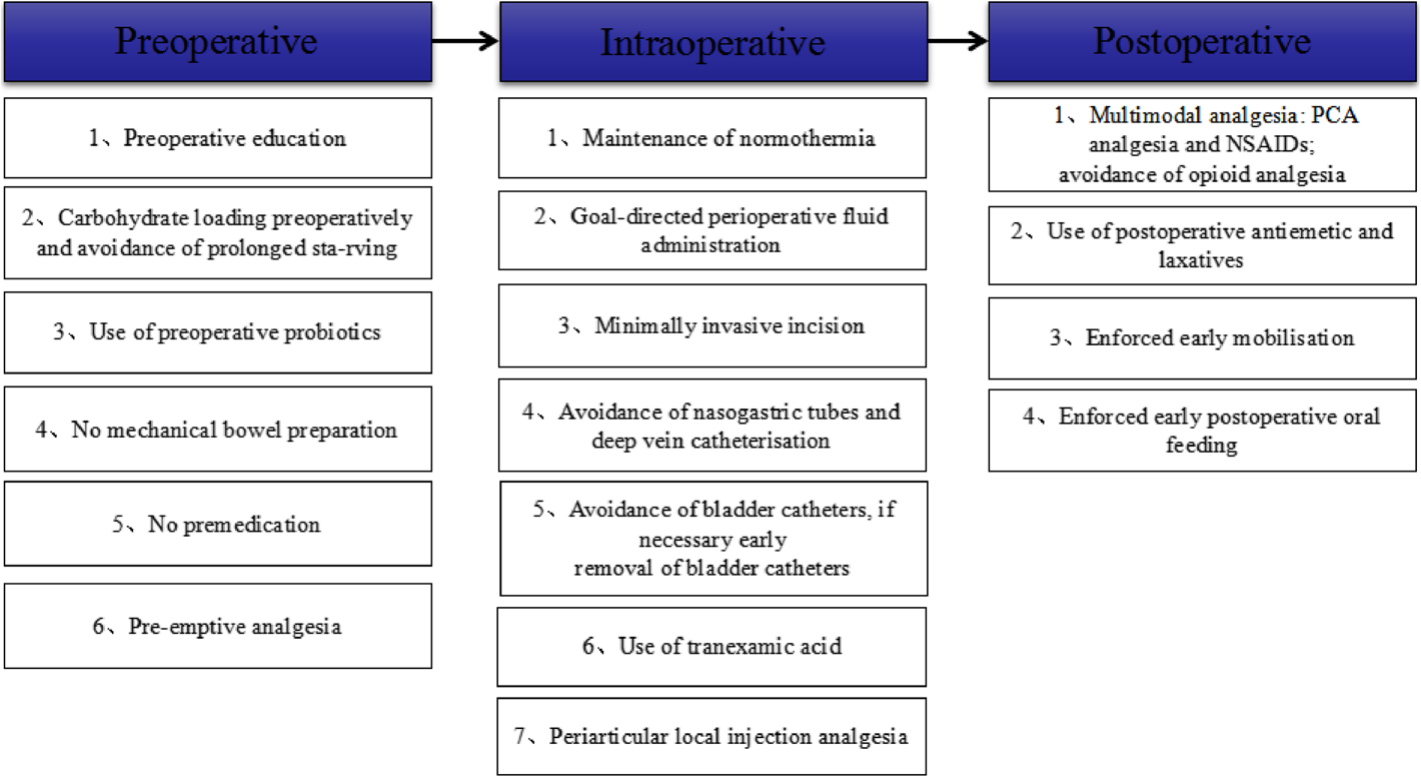
Enhanced recovery after surgery pathways

**Fig. 2:**
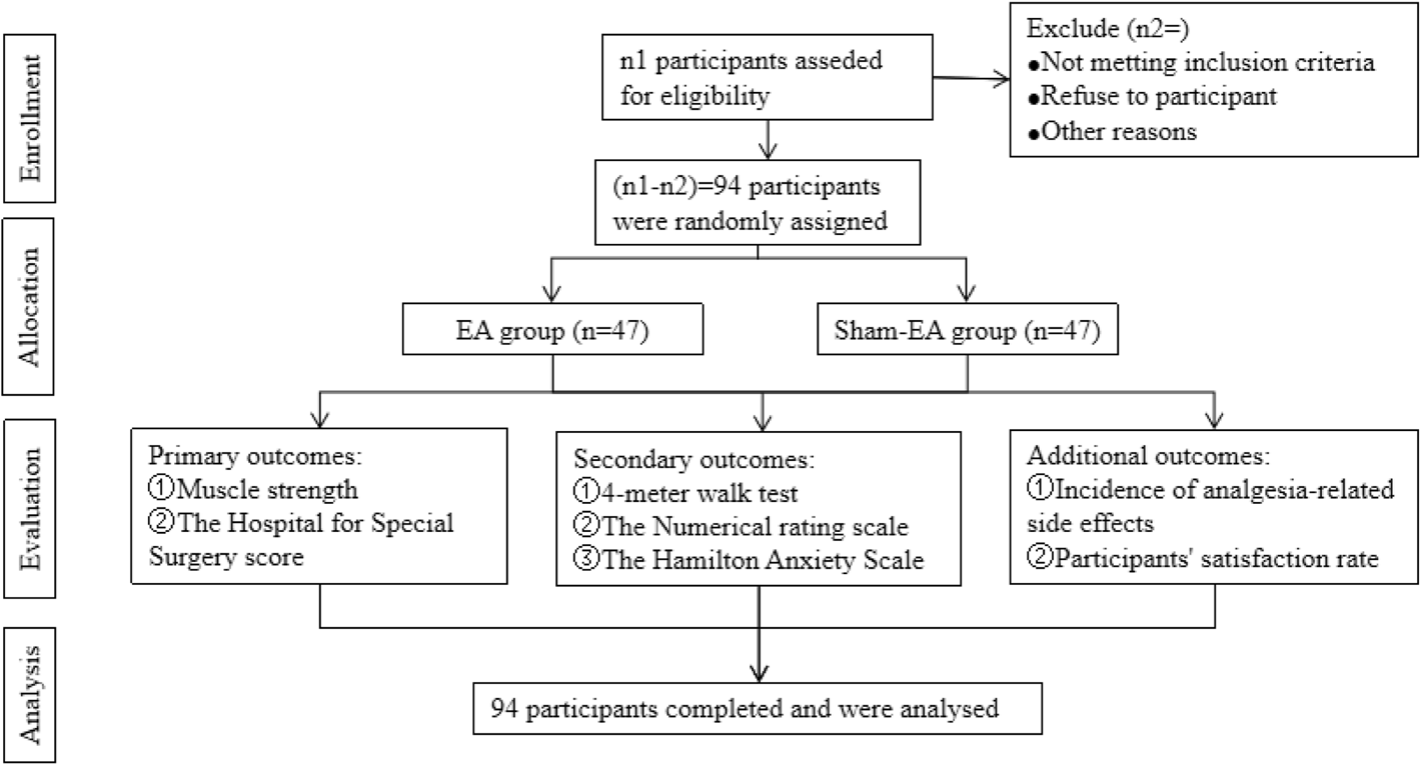
The trial flow diagram

**Fig. 3:**
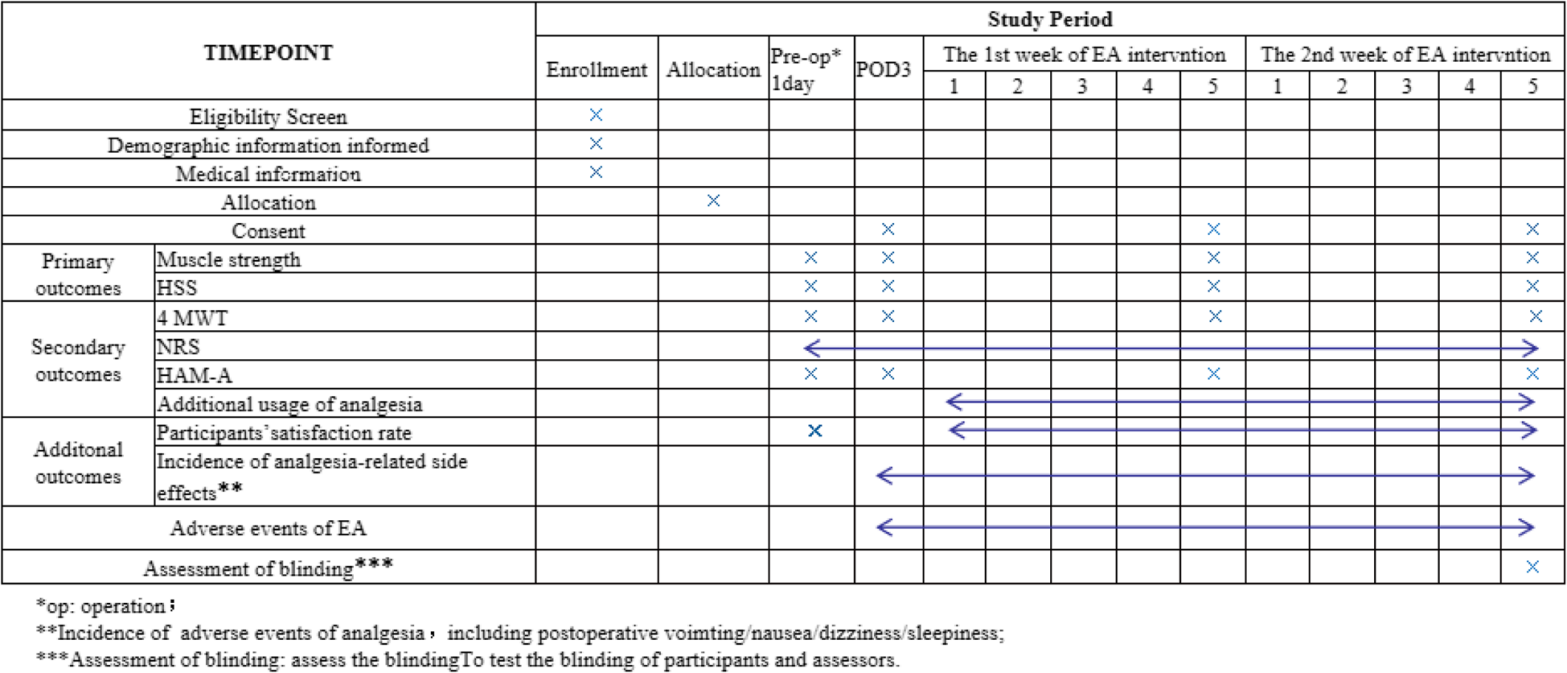
The schedule of trial enrollment, interventions, and assessments

Participant registration will be conducted between August 2020 and December 2020. Written informed consent will be obtained from all participants for inclusion in any other scientific publications. When the participants are hospitalized, a trained nurse and several physiotherapists will implement the enhanced recovery after surgery (ERAS) pathway (Fig. 1). Figure 2 presents the trial flow, which includes participant recruitment, eligibility screening, randomization, intervention, and outcome assessments. Figure 3 presents an overview of the trial design, conduct, review, and analysis. A filled-out SPIRIT 2013 checklist (Word) is presented in Additional file 1.

### Randomization and blinding

Independent research staff will use RV.3.5.1 software to generate a randomly numbered sequence for complete random grouping. A number sequence will be sealed by an independent assistant in an opaque envelope containing treatment information. Eligible and consenting patients with KOA who have undergone unilateral TKA will be randomly assigned to the EA and sham EA groups at a ratio of 1:1 (each group, *n* = 47). Only the physiotherapist in charge of EA treatment is authorized to open the envelope and enrol participants.

All the participants, physiotherapists, outcome assessors, and data statisticians will be subject to blinding. To help maximize participant blinding, a pragmatic placebo needle will be employed and a sham EA design will be applied. Unblinding before study completion will only be allowed in the case of medical emergencies or severe adverse events.

### Interventions

#### Scheme of standard analgesic use

Postoperatively, patient-controlled analgesia will be used until postoperative day (POD) 3. The used analgesic will be fentanyl (Yichang Renfu Pharmaceutical Co. Ltd., Yichang, China) at a continuous infusion rate of 0.25 μg/(kg h) and a bolus of 0.15 μg/kg with a 10-min lockout time. The locking duration of the projectile will be 10 min after administration. Further, oral celecoxib capsules (Pfizer Pharmaceutica Co. Ltd., New York State, USA) will be provided to the participants on demand between the POD 4 and POD 14. Moreover, additional analgesia consumption will be recorded.

#### Basic physical therapy

Both groups will undergo basic post-surgery physical therapy (exercise) from 24 h after TKA. The programme will be supervised by a physical therapist and involve ankle pumping exercises, hip flexion (straight leg raise), and pain-free knee mobilization in flexo-extension. The exercises will be performed daily at 30-min sessions for 2 weeks. Additionally, the participants performed ankle pumping exercises with the lower extremities elevated and respiratory exercises for the first 24–48 post-TKA hours.

#### EA group

**Fig. 4:**
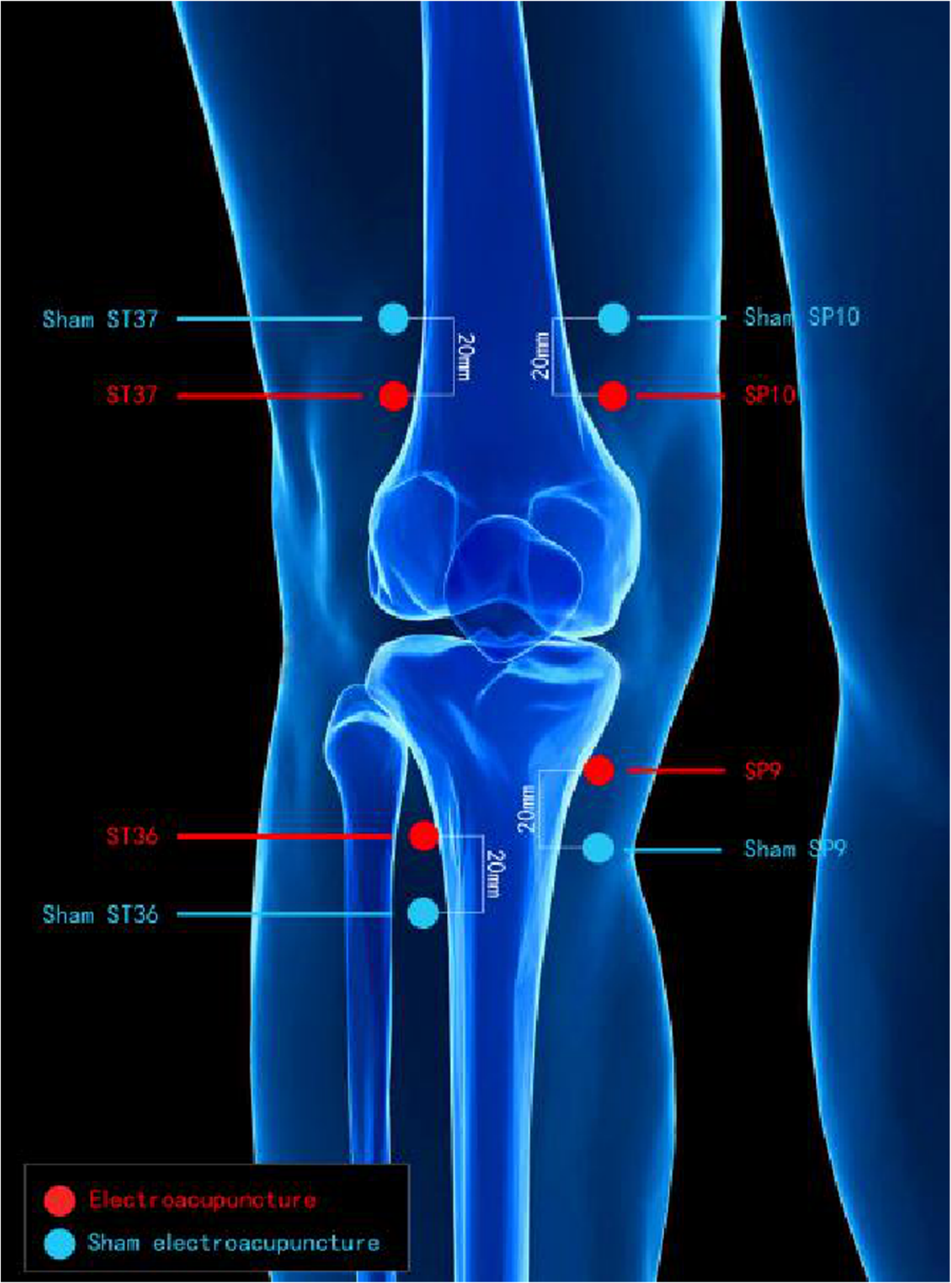
Location of acupoints for the EA and the sham EA groups

Based on the new standard of “International Acupuncture Nomenclature” [29], the LIANGQIU (Stomach 37, ST37), ZUSANLI (Stomach 36, ST36), XUEHAI (Spleen 10, SP10), and YINLINGQUAN (Spleen 9, SP9) acupoints on the surgery side will be employed (Fig. 4). After skin disinfection, adhesive pads (size 8 × 10 mm, Suzhou Medical Supplies Factory, Suzhou, China) will be placed on the acupoints through which acupuncture needles (size 0.25 × 40 mm, Suzhou Medical Supplies Factory, Suzhou, China) will be inserted approximately 3 cm into the skin (Fig. 4). After achieving de qi sensation, the acupuncture points will be stimulated using Huatuo SDZ-II EA apparatus (Suzhou Medical Supplies Factory, Suzhou, China). The intensity of the constant current square wave pulse at a frequency of 40 Hz and a pulse width of 1 ms will be gradually increased until it reached an intolerable intensity. The treatment of the EA group will involve ten 30-min sessions over 2 weeks (5 sessions per week).

#### Sham EA group

**Fig. 5:**
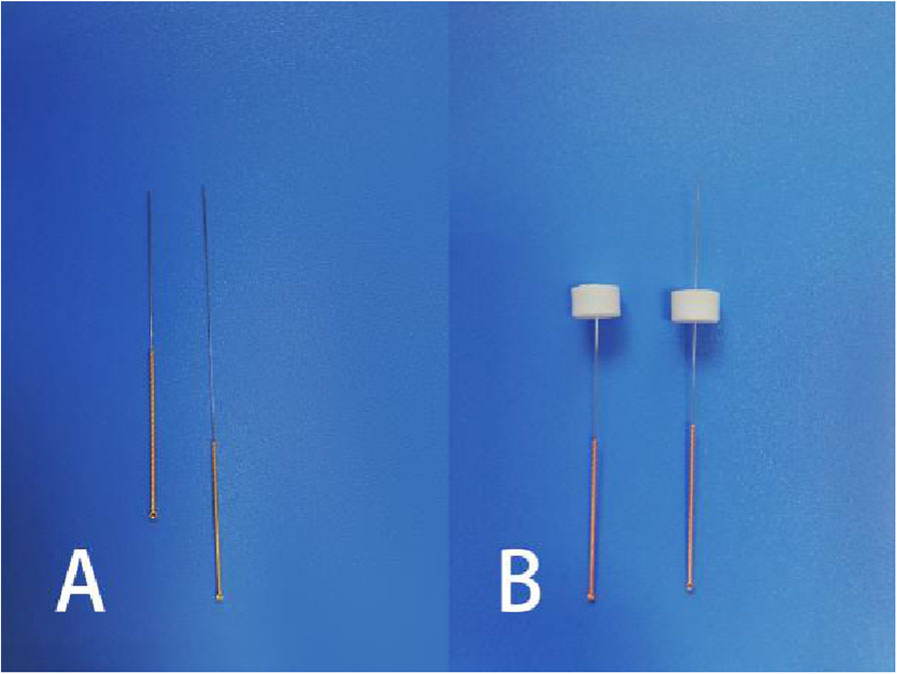
Difference of acupuncture needle and acupuncture depth between the two groups. **a** The tip of the pragmatic placebo needle (left) is blunt. **b** The pragmatic placebo needle (left) to be inserted into the sterile adhesive pads, but with no skin penetration. The acupuncture needle (right) to be inserted through the sterile adhesive pads into the skin

In the sham EA group, sham EA using a pragmatic placebo needle [30] (size 0.25 × 30 mm) will be applied on sham acupoints for 5 sessions per week. Each sham acupoint will be at 1 cun (≈ 20 mm) lateral to the acupoints (Fig. 5). The pragmatic blunt-tipped placebo needle will be inserted into the adhesive pads without skin penetration (Fig. 4). A Huatuo SDZ-II EA apparatus (Suzhou Medical Supplies Factory, Suzhou, China) will be connected to the sham acupoints through special electrode wires without electricity output. The sham needles will remain in the adhesive pads for 30 min in each session. The sham EA and EA treatment processes are similar except the former does not involve skin penetration, needle manipulation, and electric output.

To ensure compliance with treatment, participants will be required to register for treatment. Throughout the trial, the participants will be treated separately to prevent inter-participant communication. Implementing EA or sham EA will not affect usual care pathways (including the use of any medication), which will continue for both groups. There is no anticipated harm and compensation for trial participation.

### Outcome evaluations

The primary outcomes will include muscle strength and the Hospital for Special Surgery (HSS) score. The secondary outcomes will include the 4-m walk test (4 MWT), numerical rating scale (NRS), and the Hamilton Anxiety Scale (HAM-A) score. Further, additional analgesia use will be considered a secondary outcome. Additional outcome indexes will include the incidence of analgesia-related side effects and participant satisfaction rate.

The NRS score, additional analgesia use, and incidence of EA-related and analgesia-related side effects will be recorded daily. Evaluation of EA discomfort and acceptance will be conducted after the first day of EA intervention. Muscle strength will be evaluated at two time points, i.e. before surgery and at 2 weeks after EA intervention. The HSS score, 4 MWT, and HAM-A score will be evaluated at four time points, i.e. before surgery, before the EA intervention (at POD 3), 1 week after EA intervention, and 2 weeks after EA intervention.

#### Demographic/medical variables

The demographic/medical variables include sex, age, marital status, occupation, ethnicity information, education level, blood pressure, temperature, respiration, pulse, height, weight, body mass index, the combination of disease and medication, Kellgren-Lawrence classification, KOA course, and history of major operations.

#### Muscle strength

Knee muscle strength will be evaluated as the PT of knee flexion and extension using Biodex 4 isokinetic muscle strength tester (IST, Biodex Medical Systems, New York, USA). Before the test, the physiotherapist will stabilize the participant on a dynamometric chair in a 90° sitting position with the torso and thighs fixed. The machine’s power axis will be aligned with the knee rotation centre. Moreover, the participant will be asked to move the knee to measure the range of motion within those of previous tests. The physiotherapist will set the angular velocity at 60°/s and ask the participant to perform five constant flexion and extension activities. During the test, the participant will use his/her maximum strength in each flexion and extension activity to measure the PT of knee flexion and extension. The test will be independently conducted by the physiotherapist.

#### Hospital for Special Surgery score

The Hospital for Special Surgery (HSS) score was developed by John N. Insall, the father of the modern artificial knee joint. It was first used in 1976 for pre- and postoperative evaluation of four different types of knee arthroplasty prostheses and is reliable and effective [31]. The HSS score includes 10 main items for the comprehensive evaluation of the condition of participants with respect to pain, daily activity, range of knee motion, muscle strength, deformity, and instability. The score will be used to evaluate the participants’ pre- and post-TKA knee joint function to evaluate the EA effect on early rehabilitation. Its components will be further analysed to identify factors that can promote patient rehabilitation.

#### Physical performance

Walking capacity will be evaluated by a 4 MWT. The participants will be required to walk as fast as they normally would (with walking aids, if necessary) thrice along a 10-m corridor. The 4 MWT can predict health status, functional decline, hospitalization, disability onset, and mortality [32–34]. Participants will be allowed to use assistive devices (walkers and crutches) to walk. The test is reliable for post-TKA patients [35].

#### Pain

The NRS consists of a horizontal line segment similar to the visual analogue scale segment with the starting (“0”) and ending points (“10”) indicating “painless” and “extreme pain”/“the most severe pain experienced”, respectively. The participants will mark the scale to indicate their knee pain (pain recorded at rest and movement). The NRS will be employed given that it is easy to understand, allows wide adaptation, and offers minimal difficulty with operation [36, 37].

#### Perioperative anxiety

Psychic and somatic symptoms of perioperative anxiety will be assessed using the HAM-A, which consists of 14 items evaluated on a 5-point scale ranging from 0 (not present) to 4 (very severe) [38]. The scores of 0–7, 8–14, 15–19, 20–29, and 30–56 indicate no anxiety, questionable anxiety disorder, mild anxiety disorder, moderate anxiety disorder, and severe anxiety disorder, respectively.

### Additional outcomes

**Fig. 6:**
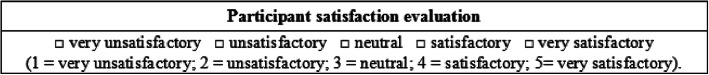
Participant satisfaction evaluation scale

Analgesia-related side effects mainly include postoperative nausea and vomiting. The participants will be asked to indicate their satisfaction rate in a scale [39] (Fig. 6).

### Adverse events of EA

EA-related complications will mainly refer to broken needles, halo acupuncture, stagnation acupuncture, hematoma at the acupuncture site, continuous post-needling pain lasting > 2 h, bleeding, etc. Moreover, we will record the adverse events during the tests: knee muscle strength using IST and 4MWT.

### Blinding assessment

**Fig. 7:**
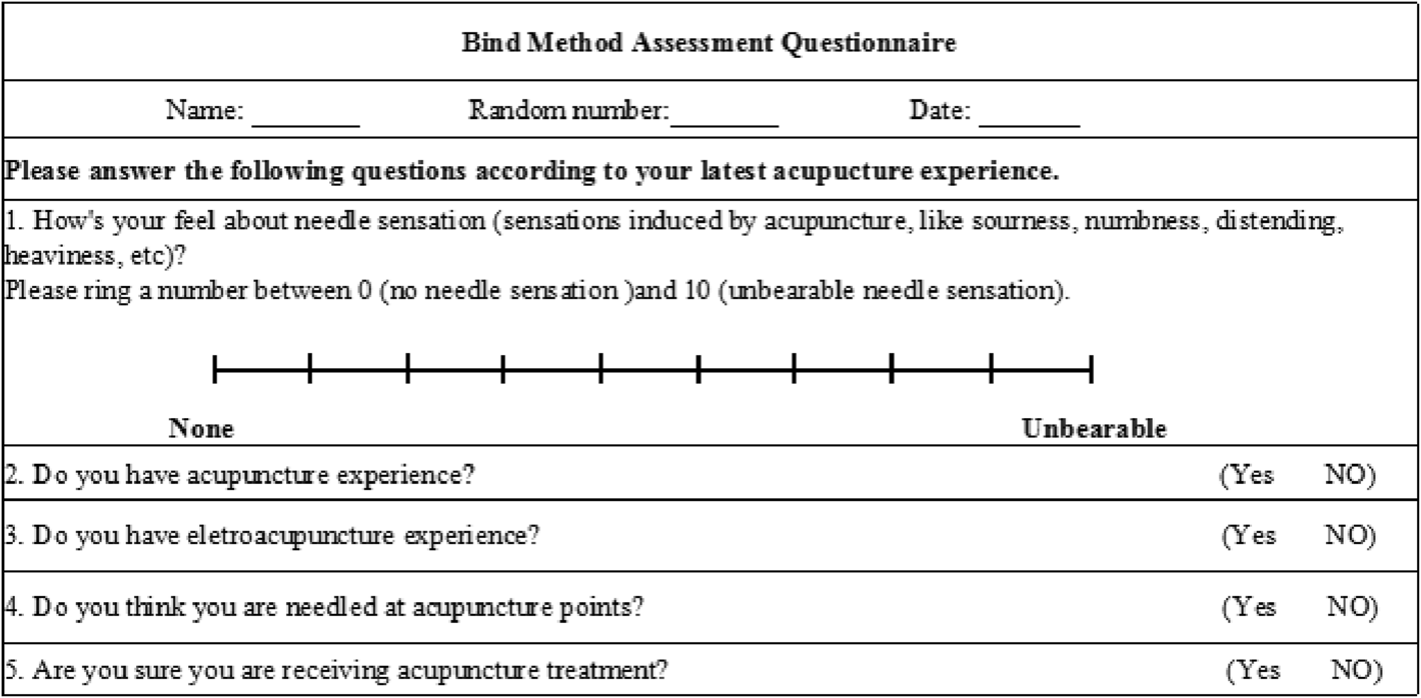
Blind method assessment questionnaire

Blinding of participants will be assessed through a questionnaire (Fig. 7) to be completed within 5 min after the last intervention. The percentage of participants from each group who thought they received true EA treatment will be recorded. Between-group differences in the participant blinding success rates will be assessed.

### Data management and monitoring

Clinical data will be carefully saved using printed and electronic case report forms (CRFs). To guarantee data quality, only outcome assessors will access the CRFs; further, they will input data and check for double entry. During the trial, Shanghai Guanghua Hospital of Integrated Traditional Chinese and Western Medicine will be responsible for making regular visits (once a week) to review trial conduct. The ethics committee of Shanghai Guanghua Hospital of Integrated Traditional Chinese and Western Medicine will monitor for protocol violations weekly. There was no conflict of interest with the sponsors or researchers. Only data statisticians will access the final trial dataset, which will only contain coded data. The safety, progress, study integrity, and design aspects will be monitored at several meetings by the involved research team.

### Statistical analysis

Based on the intention-to-treat principle, full analysis set and per-protocol sets will be used for the primary analysis. Sensitivity analysis will be performed to determine the impact of incomplete records on results. Missing data will not be imputed. Epidata V3.1 and IBM SPSS Statistics V21 will be used for data input and analysis, respectively. Complete follow-up assessments of the participants will be performed using the per-protocol sets and intent-to-treat population.

Statistical analysis will involve four aspects. First, for normally and non-normally distributed measurement data, the mean ± SD and median, respectively, will be used for the statistical description of the degree of concentration trend and dispersion. The respective between-group differences will be compared using two independent sample *T* tests and the Mann-Whitney *U* test. Second, the gender composition ratio, which is a continuous variable, will be assessed using the fourfold table chi-square test. Third, between-group differences in the ranked data will be compared using the Mann-Whitney *U* test. Finally, repeated measurement data will be compared using the generalized linear mixed models. Statistical significance will be set at *p* value < 0.05 (two-sided).

## Discussion

EA is an improvement of the traditional manual acupuncture method that involves modern electronic equipment to enhance the intensity of mechanical acupuncture stimulation for its beneficial effects, including pain relief, muscle relaxation, and circulation improvement [40, 41]. EA is effective in improving muscle strength and promoting functional rehabilitation [42, 43]. To our knowledge, the EA treatment effects on post-TKA muscle strength improvement remain to be validated by randomized and placebo-controlled trials. Consequently, this trial will aim to examine EA efficacy in improving muscle strength during the early post-TKA period compared with the sham EA. This is to provide evidence regarding clinical EA application for patients with KOA after unilateral TKA. To maximize the exclusion of placebo effects, a rigorous and methodological design will be followed, including non-acupoints, non-penetrating acupuncture, and special electrode wires with electricity output for control. Further, the participants will separately receive treatments at different times to avoid inter-participant communication and interference. The aforementioned methods will likely allow successful participant blinding and objective evaluation of the EA efficacy for post-TKA patients.

Given its decent analgesic effects, EA can reduce the post-TKA use of opioids and non-steroidal anti-inflammatory drugs. In the past several years, there has been a worldwide increase in the support for ERAS given its contribution to pain relief and functional recovery [44]. Early post-TKA EA treatment could eventually become an important component of ERAS. We believe that this trial will contribute to enhancing lower limb muscle strength after TKA, and thus provide evidence for primary TKA rehabilitation.

## Trial status

- ✓ Protocol version number: V1.1: 21 September 2019
- ✓ Date of recruitment: 1 August 2020
- ✓ Date of recruitment completed: 1 December 2020

## Supplementary information

**Additional file 1.** SPIRIT 2013 Checklist: Recommended items to address in a clinical trial protocol and related documents.

## Data Availability

The datasets and the informed consent form can be obtained from the corresponding authors upon reasonable request.

## Abbreviations

4 MWT: 4-m walk test
CRFs: Case report forms
BMI: Body mass index
EA: Electroacupuncture
ERAS: Enhanced recovery after surgery
HAM-A: Hamilton Anxiety Scale
HSS: Hospital for Special Surgery
IST: Isokinetic strength test
NRS: Numerical rating scale
KOA: Knee osteoarthritis
PCA: Patient-controlled analgesia
POD: Postoperative day
PT: Peak torque
RCT: Randomized controlled trial
TKA: Total knee arthroplasty
